# Gamification in Biomedical Science Education: The Successful Implementation of Resimion, a Scenario-Based Learning Tool

**DOI:** 10.3389/bjbs.2023.11756

**Published:** 2023-10-02

**Authors:** Jennifer E. May, Elizabeth Anderson, Dan Clark, Jonathon Hull

**Affiliations:** ^1^ Faculty of Health and Life Sciences, University of the West of England, Bristol, United Kingdom; ^2^ Resimion Ltd., Bristol, United Kingdom

**Keywords:** gamification, neurodiversity, Resimon, scenario-based learning, digital education

## Abstract

**Introduction:** Scenario-based learning and gamification have many advantages in comparison to traditional didactic teaching methods, including development of many higher-level skills such as analysis and evaluation. It is hoped that these simulations provide a real-world experience in a format accessible to students. Integration of these tools into teaching excelled during the COVID-19 pandemic, an event that completely changed education and initiated the greatest advancement in digital learning to date. We discuss our experiences using Resimion, a novel scenario-based learning tool that was adapted to biomedical science, both for teaching and assessment.

**Methods:** Our cohort included 769 students studying BSc(Hons) Biomedical Science at the University of the West of England from 2020 to 2023. Data was obtained from assessments within four different modules, two at FHEQ level 5 and two at level 6. Students were grouped based on reasonable adjustment (RA) status, including physical issues, specific learning differences and neurodiversity, with differences between student groups and assessment types analysed by ANOVA.

**Results:** Data clearly demonstrate good engagement from students utilising Resimion software, representing 18,436 student interactions in total, across both assessed and non-assessed activities. RAs of any type did not alter submission rates (*p* = 0.53) or student outcome in any of the assessment types analysed. However, submission rates for Resimion assessments were notably higher than for other assessment types (*p* = 0.002). Whist outcomes were not significantly different, students with RAs did take significantly longer to complete the Haematology and Transfusion assessments (*p* = 0.0012). Specifically, neurodiverse students and those with specific learning differences used on average 81% of their allocated time, students with other RAs used 76%, whereas students without RAs used just 56% (*p* ≤ 0.0001), highlighting the appropriate adjustment of extra time provided for these students. It was further observed that 1.3% of Resimion activities undertaken by students utilised the in-built inclusivity features in the software. Both students with known RAs, and those without, utilised these features, therefore also aiding students without a formal diagnosis.

**Conclusion:** The scenario-based learning tool Resimion was successfully integrated into the teaching of biomedical science and provided an engaging platform for students, with comparable results to other traditional assessment types.

## Introduction

The concept of gamification or scenario-based learning (SBL) has been present since the early 2000s, with the term “gamification” first coined in 2008 [1]. This acts as a potential alternative to traditional pedagogical or assessment techniques, developing many higher-level skills such as analysis and evaluation. It is the hope that integration of these games will improve engagement, both inside and outside of the classroom [2, 3], allowing students to engage with their education in novel ways, through case-based learning and interactive assessment types [4]. Using SBL has already shown promise when compared to didactic teaching [5, 6]. Whilst educators are often keen to minimise didactic teaching, stimulating engagement through interactivity, it is challenging to find appropriate interactive software to develop and test skills such as critical thinking [7]. This was most obvious during the COVID-19 pandemic, an event that completely changed education. The integration of technology into teaching was rapid, however students often did not find this as engaging as traditional methods [8]. Furthermore, the evidence on the efficacy of gamification is mixed. Many authors support the notion that the higher engagement leads to better outcomes for students. However, it was argued that gamification may not be applicable to all curricula (reviewed in [9]). Mekler et al. [10] failed to find improvement in student grades with the integration of gamification. The improved student motivation and engagement needs to be coupled with factors such as autonomy, competence, and relatedness—all factors of the self-determination theory [11]. Whilst students must commit to engaging with their education, educators must commit to providing an engaging atmosphere, simulating the work environment and demonstrating the relevant skills or competencies required for their profession. In some respects, these are the underlying tenets of the Institute of Biomedical Science (IBMS) accreditation for degrees (QAA Benchmark statements) [12].

The paucity of gamification or SBL resources to educate and assess biomedical science contrasts with the depth of resources available for clinical education [13–18]. Biomedicine is a complex topic relying on diverse forms of expertise, discussing the interaction of multiple specialisms that occur within the hospital laboratories. It is often difficult for students to appreciate the specialist biomedical content, and subsequently understand how this information integrates with other specialisms. This holistic understanding, enabling integration and application of knowledge is essential, but is difficult to assess with current techniques. Gamification and SBL, and assessment via these methods, has the potential to provide learners with opportunities to be involved in the complete clinical case, gaining experience in a patient-free, risk-free game [19]. In a level 6 module at our institution teaching haematology and transfusion science, we wanted to assess the students capacity for interpretation and decision-making, rather than just knowledge recall through multiple choice questions or discrete essay questions. Traditionally, our institution has used case studies to demonstrate the integration of specialisms and allow application of knowledge. However, it can be challenging to engage students when these are paper-based. Furthermore, it is difficult to provide meaningful personalised feedback to the large cohorts we experience. This led us to integrating Resimion, a novel software designed for SBL, which had not previously been used in the biomedical/biological Science field, enabling case-study based scenarios to be developed and utilised both for teaching and assessment.

Resimion is a platform for applied learning, enabling learners to work through problem- or scenario-based activities. Utilising gamification to increase interest and deepen learning, scenarios can be run competitively with a leaderboard for anonymous peer comparisons, or individually, challenging students and providing opportunity to apply knowledge and learn from their decision-making. For example, students can be presented with background case study information for a patient and can then make informed decisions to “perform” relevant lab investigations, subsequently interpreting and analysing this data to make decisions or reach conclusions. This informal gamified learning environment can improve student motivation and encourage participation and ownership. As Resimion can be accessed via web-browser or mobile phone App it is ideally suited for use in today’s increasing digital learning environment, both for applied SBL inside and outside of the classroom, and for formal assessment.

Understanding elements of game design and human motivation, gamification can provide novel techniques to engage and assess students. Engagement, education and assessment are no longer confined to classic educational environments. Whilst the integration of gamification and SBL into teaching is increasing, it is important to note that there is a broad lack of empirical evidence assessing gamification outcomes in certain groups (e.g., neurodiverse students and those with specific learning differences). It is the hope that SBL integration will lead to improved outcomes in students who typically underperform in standard assessment styles, such as those with neurodiversity or minority ethnicities. In this work, we detail the use of gamification and SBL to assess learner progress and attainment in the IBMS accredited BSc(Hons) Biomedical Science programme at the University of the West of England. Whilst we also extensively used Resimion for gamification and SBL within non-assessed activities throughout our teaching (for example, competitive leaderboard-based games in taught sessions, and non-assessed individual scenarios), this paper focuses on measuring the effective use of Resimion for assessments.

The aim of this work was to analyse student engagement and feedback using Resimion, alongside comparing overall student outcomes from Resimion-based assessments with other assessment types. A further aim was to assess any impact of utilising Resimion on neurodivergent students and those with disabilities, by comparing engagement and assessment outcomes utilising such SBL and gamification, alongside traditional forms of assessment. It is proposed that the trends observed in our student datasets will inform pedagogical teaching methods. This work describes for the first time the implementation of Resimion in a Biomedical Science programme.

## Methods

### Student Cohort

Our student cohort includes 769 individuals studying BSc(Hons) Biomedical Science during the period of September 2020 to June 2023 at the University of the West of England. Data were collected from the second-year (FHEQ level 5) modules “Blood Science” (185 students over 2 years) and “Studies in the Biology of Disease” (314 students over 2 years) covering all biomedical science disciplines, alongside the third-year (FHEQ level 6) modules “Clinical Biochemistry” (55 students) and “Haematology and Transfusion Science” (215 students over 3 years). Only “Studies in the Biology of Disease” is classed as a core module, which students must complete, with the remainder classed as “optional” modules. Students select from several “optional” modules, therefore there may be overlap between our cohorts enrolled on each module, depending on the module selections made by students.

### Use of Resimion and Assessment Types

Within this study, data was collected from Resimion-based activities utilised for both assessed and non-assessed purposes. Within the second-year “Studies in the Biology of Disease” module and third-year “Haematology and Transfusion Science” module, students were given access to interactive activities both within and outside of class, including case studies, quizzes and multiple-choice questions (MCQs), to aid their learning and provide opportunities for application of knowledge. Additionally, both of these modules used Resimion for assessment, utilising quizzes for “Studies in the Biology of Disease” and longer case studies and randomised MCQs for “Haematology and Transfusion Science.”

Alongside Resimion-based assessments, we analysed other assessment types within the same modules and in two other modules undertaken by the same year group in the BSc(Hons) Biomedical Science programme. The assessment types analysed within this study varied across the four modules studied, and additionally underwent changes during our study period due to the impact and restrictions imposed during the COVID-19 pandemic. Details of all assessment types for each of the four modules are listed in Table 1.

**TABLE 1:** Summary of assessment types used across four modules in the BSc(Hons) Biomedical Science programme at the University of the West of England.

Assessment	Brief description
On campus, timed exam	Studies in the Biology of Disease (level 5): Students completed a 3 h unseen timed exam under controlled conditions on campus. These questions represented multiple specialisms relating to a specific case study. The case study was released 1 month before the exam without associated questions. Students were allowed to bring an annotated version of the case study into the exam with them
	Haematology and Transfusion Science (level 6): Students completed a 2 h unseen timed exam under controlled conditions on campus. One essay question (from a choice of 3) and one extended case study were completed. Students were allowed to bring in an A4 closed box file of notes
	Clinical Biochemistry (level 6): Students completed a 3 h unseen timed exam under controlled conditions on campus. There were six case studies with each lecturer providing 2 from their taught content. Students were allowed to bring in an A4 closed box file of revision notes
	Reasonable adjustments: For all exams, extra time was available (up to 50%) for students with reasonable adjustments. Further adjustments were available such as individual rooms, facility to type answers, dependent on individual needs
	This style of exam could not be completed during major COVID restrictions and was substituted with a 24 h online exam, but has been reintroduced for academic year 2022–23
Online, 24-h exam	Students accessed an exam paper online through Blackboard. Papers were available for a 24 h period, with answers limited by word count rather than by a shorter time period. This approach was used for all modules during 2019–20, 2020–21 and 2021–22 [and is still in place for some modules where assessments are not stipulated by professional bodies, for the purposes of this paper the “Blood Science” module (FHEQ level 5)]
	Blood Science (level 5): Students answer 2 questions from each of three sections (from a choice of 4). Word limit of 250 words per question (1500-word limit in total). Reasonable adjustments: No extensions or extra time was available for the 24 h exams
Media clip	Blood Science (level 5): Within the Blood Science (level 5) module, the coursework assignment consists of students completing a 5 min video on a blood science-based point-of-care test of their choice. Students must be visible in the clip throughout, and must include certain content, such as comparison of their test with competitors, and explaining the underlying principles of the test. Reasonable adjustments: 5 day grace period, extensions were available
	Clinical Biochemistry (level 6): A 5 min summary video on a specific disease commonly diagnosed through the Clinical Biochemistry biomedical specialism. Students must be visible in the clip throughout, and must include certain content, such as pathophysiology, diagnostic tests, and future research. Reasonable adjustments: 5 days grace period, extensions were available
Reflective coursework	Studies in the Biology of Disease (level 5): A reflective short answer essay similar to the Continual Professional Development available in the Biomedical Scientist journal (Supplementary Material USSKAT-30-2 CW2 CPD). Reasonable adjustments: 5 days grace period, however no extensions were available
Written case study	Studies in the Biology of Disease (level 5): One patient case study with 6x 250-word questions, covering each biomedical specialism (Supplementary Material USSKAT-30-2 CW3 MARIANA). Reasonable adjustments: 5 days grace period, however no extensions were available
Resimion MCQs and case study	Haematology and Transfusion Science (level 6): Coursework comprises three assessed tasks using Resimion. These are available for 24 h after each practical class and are based on theory and practice from each section of the module (haematological malignancies, haemostasis and transfusion). Students have 40 min once they open the assessment to complete three randomly allocated MCQs and a longer case study (with randomised elements). Reasonable adjustments: No extensions or 5 days grace period available as a timed assessment. Reasonable adjustments for extra time (up to 50%) were automatically incorporated for eligible students
Weekly Resimion quizzes	Studies in the Biology of Disease: After every lecture, a Resimion was released that assessed the knowledge in the field that related to the lecture. This could be in the form of MCQs, case studies, blood typing panels, or picture quizzes. These quizzes had a 15 min time limit; however, students could have unlimited attempts. Reasonable adjustments: There are no reasonable adjustments for this submission

Level refers to the FHEQ level defined by the QAA Qualification Framework for Higher Education.

The “Blood Science” and “Clinical Biochemistry” modules did not utilise Resimion for teaching or assessment, but were included in this study for comparison of student performance across other styles of assessment.

### Reasonable Adjustments/Disabilities

Data regarding status of “reasonable adjustments” for individual students was provided by the University’s Disability Services.

All data have been anonymised within this study, with students grouped into categories according to their disability or reasonable adjustment requirements. Students were categorised as “no RA” if they do not have disability or eligibility for additional requirements recorded on their university record. Our institution lists students as eligible for “reasonable adjustments” if they have any declared condition, disability or learning need. Often additional information is given for each RA detailing the exact adjustments needed, for example, physical adjustments, extra time, or measures to support mental health conditions. For the purposes of this study, we grouped all students as “RA” if they had reasonable adjustments recorded, whether permanent or temporary, including both physical and mental health conditions. We subsequently separately grouped students who had an RA recorded, which noted a specific learning difference (SpLD) (such as dyslexia, dyspraxia, dysgraphia) or neurodiversity (ND) [including autism spectrum conditions or attention deficit (hyperactivity) disorder], as an SpLD/ND category.

### Statistical Analysis

Statistical analysis was performed in GraphPad Prism version 9. One-way ANOVA with Tukey’s multiple comparisons test was used to examine differences between student groups, with *p* < 0.05 considered to be statistically significant. A two-way ANOVA was used to analyse differences in submission rates for each student cohort according to assessment type.

### Ethical Approval

The data from the modules were anonymised prior to analysis. All data were collected anonymously as part of routine monitoring and evaluation of the modules. Students consented to providing feedback on Resimion through anonymous data collection using Mentimeter for routine module evaluation. This work is part of an ongoing project at UWE and has been given ethical approval by the University ethics committee (Ref No. HAS.23.06.133).

## Results

This study included 769 students over a 3 years period, undertaking the IBMS accredited Biomedical Science degree at the University of the West of England. The work represents the outcomes on four modules: Blood Science (FHEQ level 5), Studies in the Biology of Disease (level 5), Clinical Biochemistry (level 6), and Haematology and Transfusion (level 6). The types of assessments undertaken were wide-ranging, including a 24 h open book exam, 2 or 3 h open book on campus exams, case-study essays, a reflective essay, creation of media clips, and Resimion assessments (Table 1). These provide a range of assessment features that challenge students (Table 2), and assess a number of different skills, in addition to specialist biomedical science knowledge.

**TABLE 2:** Comparison of features within assessment types used across four modules in the BSc(Hons) Biomedical Science degree at the University of the West of England.

Assessment features	On campus, timed exam	Online, 24-h exam	Media clip	Reflective coursework	Written case study	Resimion MCQs and case study	Weekly Resimion quizzes
Provides instantaneous feedback						✓	✓
Provides personalised detailed feedback			✓	✓	✓	P	
Emphasises understanding	✓	✓	✓	✓	✓	✓	P
Analyses choice utilisation			P	✓	✓	✓	✓
Monitors engagement						P	✓
Assesses knowledge of biomedical science	✓	✓	✓	P	✓	✓	✓
Promotes self-evaluation				✓		✓	
Accommodation of reasonable adjustments	✓		✓			✓	
Student choice of assessment topic			✓	P			
Student self-selection of accessibility features						✓	✓
Minimises assessment offences	✓		✓	✓		✓	P
Time restrictive	✓	P	P			✓	✓
Creates an individualised experience			✓	✓		✓	✓

✓—represents positive response for that feature. P—represents partial response for that feature.

For analysis of Resimion data, the work represents 18,436 student interactions in total within Resimion software. This incorporates both assessed and non-assessed activities, including synchronous (in-class), and asynchronous (independent) interactions.

### Students Interaction With Resimion Was Positive and Comparable for Neurodiverse and Neurotypical Groups

Our data demonstrate good engagement (defined here as students attempting a specific activity) from students utilising Resimion in their learning, both within the classroom and as an independent study aid. In class, students reported both enjoyment and benefits in terms of opportunity to apply the theory they had learnt. Final year students were given the opportunity to undertake asynchronous case studies through Resimion following lectures, with 74% of students utilising this opportunity. We have also observed that 1.3% of Resimion activities undertaken by students utilised the in-built inclusivity features in the software. This use of self-modified inclusivity features represents 517 instances of student interaction, benefitting students potentially not officially recognised as needing reasonable adjustments. From polling students anonymously in class, we have observed that many students suspect they are neurodiverse or have SpLDs, but lack a formal diagnosis (data not shown). Therefore, self-modified features will be invaluable to these students. The most common inclusivity features used by students were alterations to panel or colour themes (*n* = 358), background (*n* = 318), alongside enabling of the text-to-speech function (*n* = 159) (Table 3).

**TABLE 3:** Percentages of quizzes/case studies during 2022/23 where students self-enabled inbuilt accessibility features in Resimion.

Accessibility feature	Percentage of total case studies/quizzes where feature was utilised: (%)
Background colour changes (i.e., to change contrast)	0.8
Altered panel colours or alternative colour theme selected	0.9
Text colour changes	0.12
Text-to-speech enabled	0.4
Any accessibility feature enabled	1.3

*n* = 39,823 case study/quiz student interactions in total across the modules “Haematology and Transfusion Science” and “Studies in the Biology of Disease.”

Feedback from students has been extremely positive regarding the use of Resimion, with many requesting increased use of this approach both within these modules, and more widely across the degree programme (Table 4). We also sought to gain feedback from neurodiverse students on our modules regarding their experience with using Resimion, with comments received including:

“I thought Resimion was simple and easy to use. The time limit and negative marking was stressful in the moment of completing the case studies, but the practice Resimions made it easier. Overall, I really enjoyed the coursework and preferred it to written coursework”

“Resimion also relieved coursework stress as it was quick to complete. I’d pick Resimion over a paper-based coursework”

“Very helpful, because you implement knowledge based on real-life scenarios. I love more practical engagements to learning”

“It didn’t feel like coursework, there wasn’t any stress or worry about it, as well as it actually tested other things than memory and ability to reference”

**TABLE 4:** Qualitative feedback from students of their experiences of using Resimion.

Theme	Feedback
Engagement/interest/enjoyment	• Resimions are great
	• It did not feel like coursework, there was not any stress or worry about it, as well as it actually tested other things than memory and ability to References
	• Coursework is well laid out and easy to understand
	• I would like to have more practice resims, and extra sessions on them for every subject change
	• I enjoyed the coursework, however would like more insight into how the points are scored. The combination of multiple choice questions and case study, I thought, worked really well on this module
Time management	• It is really useful to have it broken up into topics and because of this I feel well prepared for the exam too!
	• I preferred that it was broken down into chunks, also relieves pressure if you end up messing one up
	• The smaller chunks were definitely preferable to a large piece, it made it a lot less stressful compared to other modules
	• I liked that it was over three separate cases and I think they were spaced out really well. I found the inputting of some of the final diagnosis difficult in a way that haemophilia had to be entered to unlock haemophilia A, for example, but overall enjoyed
	• The coursework is broken down and interactive
Assessment compared to traditional types	• Not neurodiverse but enjoyed having short, case study based CW rather than essay based
	• Resimion CW—is a break from long essay CW in other modules
	• Whatever happens, do not change the style of cw. Make it tougher if required, but do not remove it all together
	• Keep the cw the same. I do not mind altering the number of resims to make it more difficult. But the amount of essays this year has me dead. Heam cw is probably the best and realistic
Application of knowledge	• I really enjoyed the CW been split into three Resimions, really helped understand the lecture more. I believe it should be kept as it gives a fair chance to students to do well, understand the lab tests
	• The coursework is great! It does not take up a huge amount of time but really solidifies the content taught in lectures and compliments the practicals
Interactivity/practical application	• I really enjoyed the case study aspect rather than a written assignment because you engage practically with knowledge you have been given
	• I really enjoyed the coursework. Very interactive and allowed me to bring out all I’ve learnt. Great for memory training too
	• Very helpful, because you implement knowledge based on possible real-life scenarios. I love more practical engagements to learning

Feedback was sought regarding their experience in both non-assessed activities and assessments within the Haematology and Transfusion (FHEQ level 6) module during the 2022–23 academic year.

**FIGURE 1:**
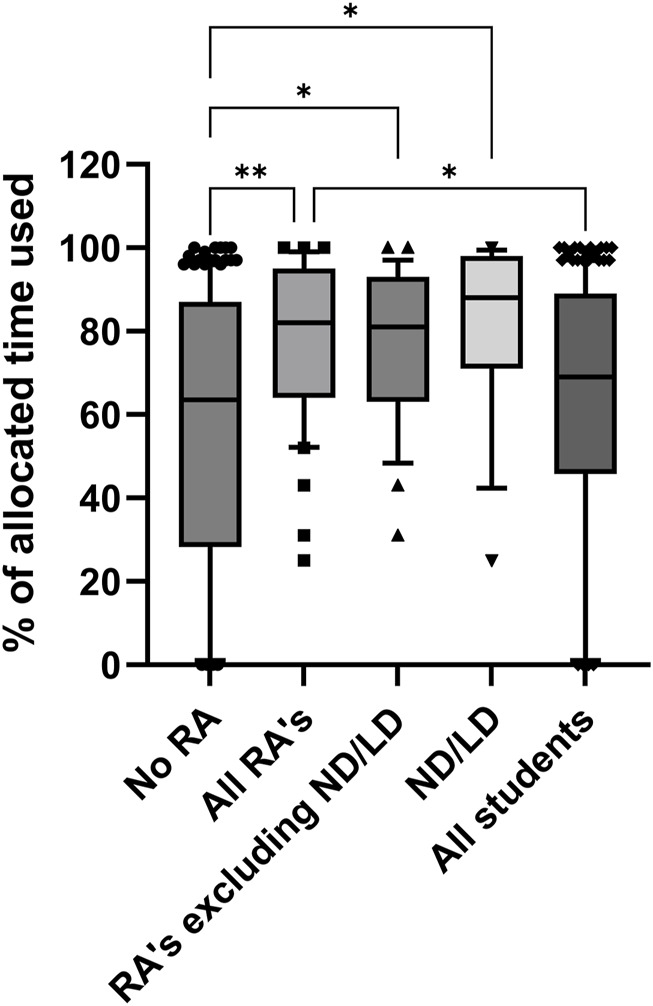
Percentages of available time utilised by students for assessed Resimion case studies in the Haematology and Transfusion Science module. *n* = 210 student case study interactions in total, comprising up to 3 assessments per student during 2022–23. Some students with RAs or ND/LD had 25 or 50% extra time allocated, depending on their specific adjustments in place. Abbreviations: RA, reasonable adjustment recorded; ND/LD, student record indicates neurodiversity or diagnosis of a specific learning difference. Data plotted as 10–90th centile, with middle 50% represented by each box, and outliers shown as symbols. *p < 0.05, **p < 0.01.

**FIGURE 2:**
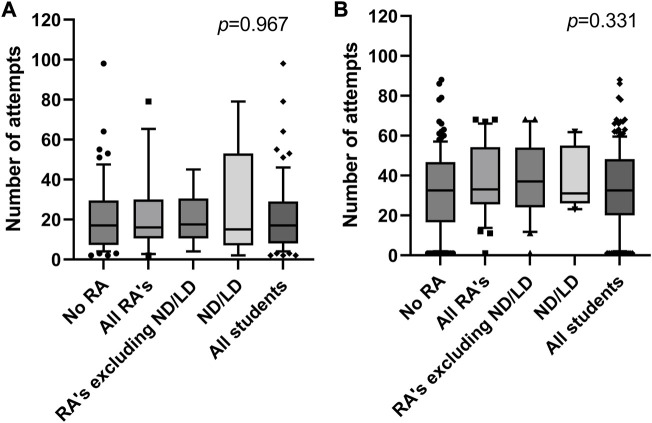
Distribution of the number of interactions with Resimion per student over the academic year 2022–23, including both assessed and non-assessed activities. **(A)** Students enrolled on the Haematology and Transfusion Science module (*n* = 69), comprising 56 students with no RA, 13 students with RAs, *n* = 8 RA excluding ND/LD, *n* = 5 ND/LD students. **(B)** Students enrolled on the Studies in the Biology of Disease module (*n* = 163), comprising 136 students with no RA, *n* = 27 students with RAs, *n* = 15 RA excluding ND/LD, and *n* = 12 ND/LD students. Abbreviations: RA, reasonable adjustment recorded; ND/LD, student record indicates neurodiversity or diagnosis of a specific learning difference. Data plotted as 10–90th centile, with middle 50% represented by each box, and outliers shown as symbols.

Importantly, neurodiverse and students with SpLDs or RAs did not have differing outcomes to students without RAs, in Resimion-based assessments (or any other assessment type that we analysed). However, it is of note that whilst outcomes did not differ when assessed through Resimion, how these students interacted with the software did vary at times. For example, within the Haematology and Transfusion Science module Resimion assessments, it was noted that students with RAs took significantly longer to complete the assessments (*p* = 0.0012). Specifically, neurodiverse students and those with SpLDs used on average 81% of their allocated time (up to 50% additional time), versus 76% for students with other RAs (excluding those students with RAs for SpLDs or neurodiversity), and 56% for students without RAs (*p ≤* 0.0001, Figure 1). This difference was only for assessed Resimion activities, with no significant differences observed between any cohort in terms of time taken per attempt (data not shown) or number of interactions with non-assessed activities (*p* = 0.967, Figure 2A). It was noted that the number of interactions for each student across the module varied widely in all student groups, ranging from minimal engagement up to 98 interactions (Figure 2A). Similarly, in the Studies in the Biology of Disease module, although Resimion activities were assessed, these were formatted differently, with all students having unlimited attempts to reach a pass mark (80%) within a 15 min timed quiz. Comparable results were observed in terms of time taken, with ND/SpLD students taking 6.1 min on average compared to 4.3 min for students with other RAs, and 4.9 min for students without RAs. Number of attempts students used to complete the quizzes were also comparable across the groups (*p* = 0.331, Figure 2B).

### Students With Neurodiversity Perform Comparably to the Main Student Cohort Regardless of Assessment Type

“Learning and assessment experiences should be diverse to both reflect the variety of the subject and to increase accessibility for all” (QAA Benchmark Statements, 2023) [12].

**FIGURE 3:**
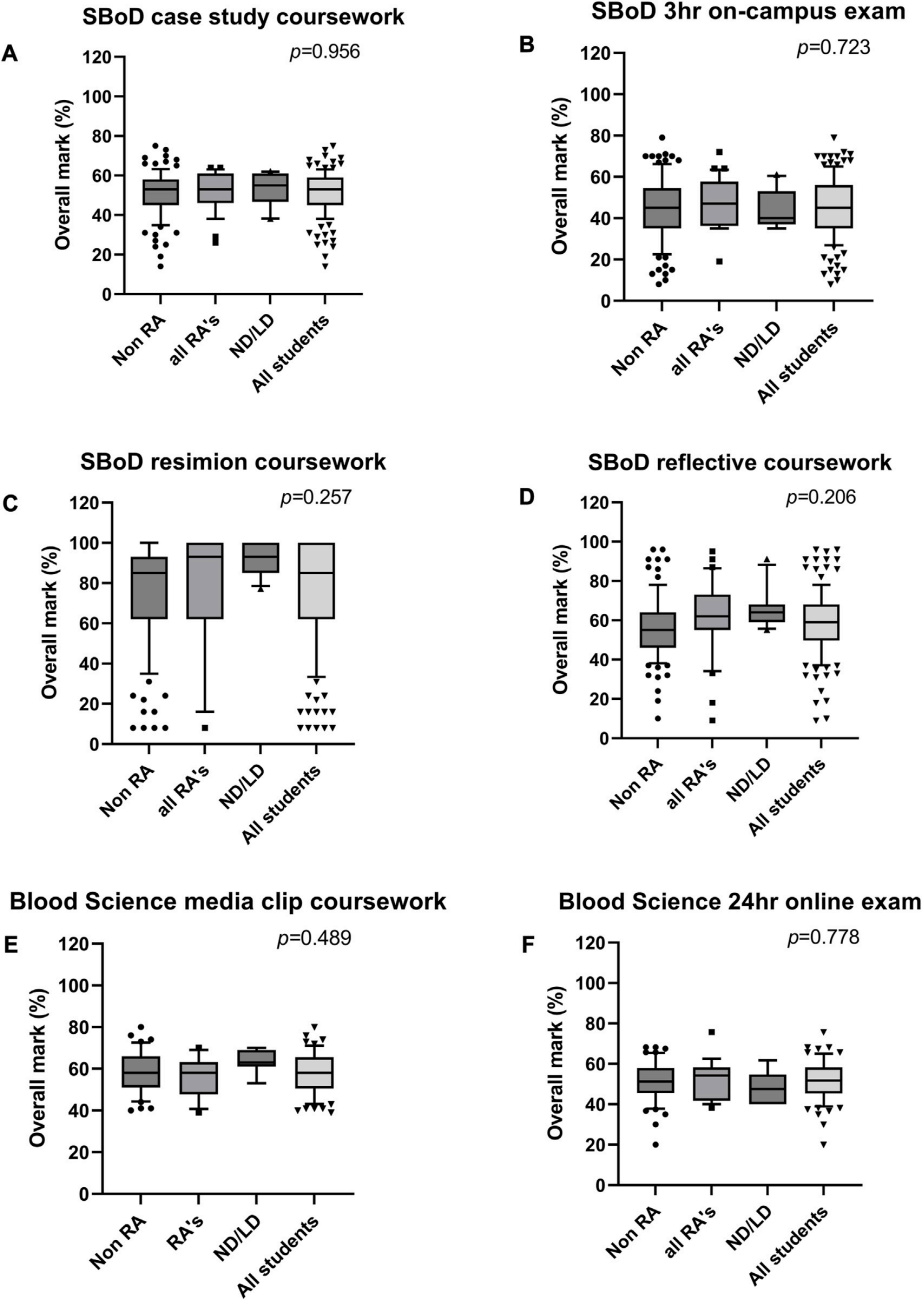
Distribution of marks achieved by students across two level 5 modules, during the academic year 2022–23. **(A–D)** Distribution of marks within assessments in the Studies in the Biology of Disease (SBoD) module, including a paper-based case study **(A)**, a 3 h on campus exam **(B)**, quizzes undertaken in Resimion **(C)**, and a reflective piece of writing **(D)**. **(E,F)** Distribution of marks within assessments in the Blood Science module, including production of a 5 min media clip **(E)** and a 24 h online exam **(F)**. SBoD module includes 155 students total, comprising 116 without RA, 39 with RAs, of which 11 are LD/ND students. Blood science module includes 87 students total, comprising 65 without RA, 22 with RAs, of which 9 are LD/ND students. Abbreviations: RA, reasonable adjustment recorded; ND/LD, student record indicates neurodiversity or diagnosis of a specific learning difference. Data plotted as 10–90th centile, with middle 50% represented by each box, and outliers shown as symbols.

**FIGURE 4:**
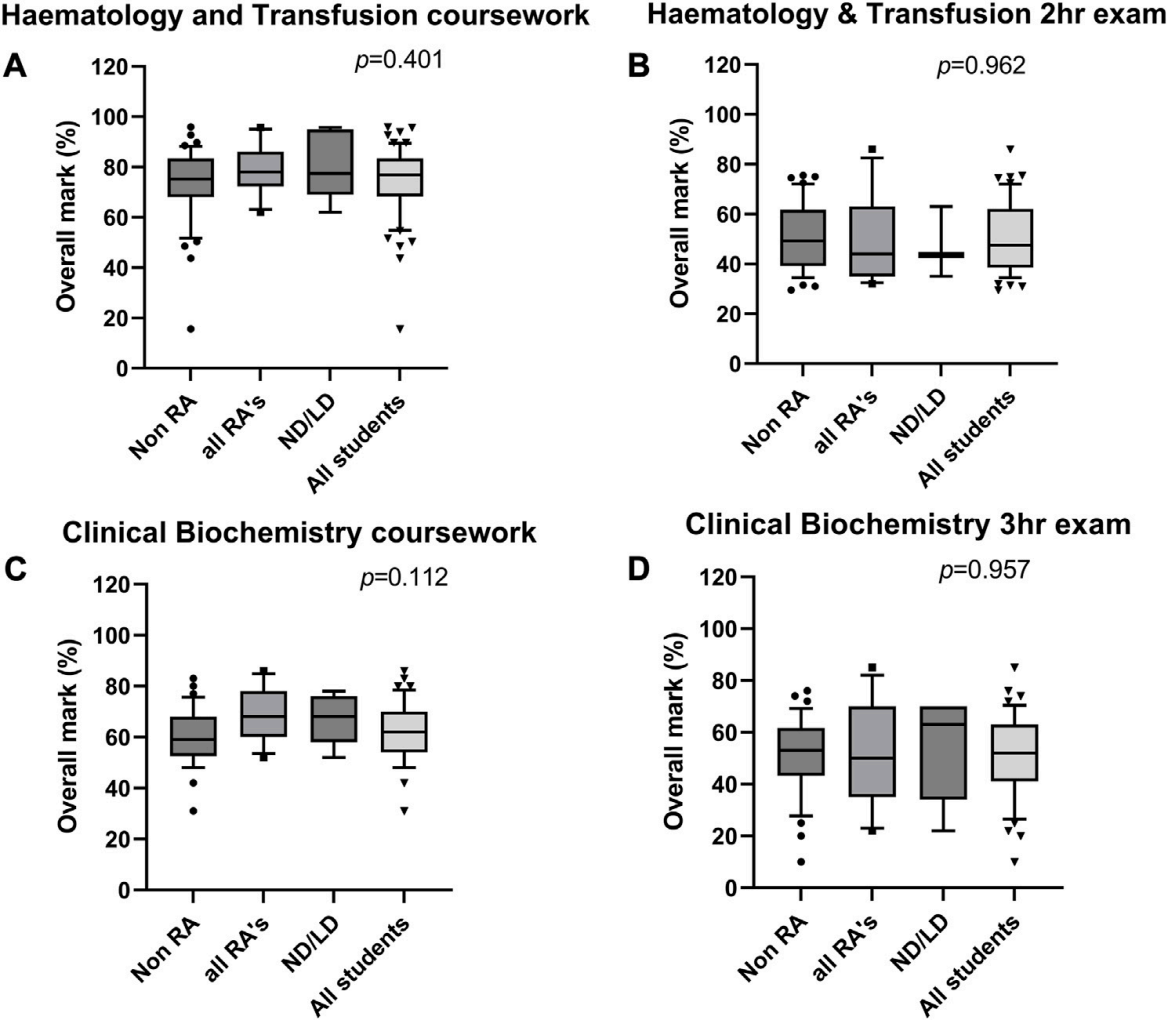
Distribution of marks achieved by students across two level 6 modules, during the academic year 2022–23. **(A,B)** Distribution of marks within assessments in the Haematology and Transfusion Science module, comprising Resimion-based case study coursework **(A)**, and a 2 h on campus exam **(B)**. **(C,D)** Distribution of marks within assessments in the Clinical Biochemistry module, comprising production of a media clip for coursework **(C)** and a 3 h on-campus exam **(D)**. Haematology and Transfusion Science module includes 65 students total, comprising 51 without RA, 14 with RAs, of which 5 are LD/ND students. Clinical Biochemistry module includes 55 students total, comprising 42 without RA, 13 with RAs, of which 6 are LD/ND students. Abbreviations: RA, reasonable adjustment recorded; ND/LD, student record indicates neurodiversity or diagnosis of a specific learning difference. Data plotted as 10–90th centile, with middle 50% represented by each box, and outliers shown as symbols.

It is important for students to experience a range of assessment types in order to fully assess skills and competencies required. However, it is also important to ensure student performance is comparable for cohorts that may struggle with certain assessment types, such as the neurodiverse, those with SpLDs and students with RAs. The assessments presented in this work represent a range of submissions across levels 5 and 6, and demonstrate that there was no difference in outcome between any of the groups assessed. The presence of specific learning differences, neurodiversity, or reasonable adjustments did not impact the student outcome of any assessment interrogated by this study (*p* > 0.05) (Figures 3, 4).

The figures presented here represents the 2022/23 cohorts for the four modules described. Further analysis of the last 3 years of data for these modules, where available, has shown the same trend, with no differences between any student cohorts observed (data not shown) (*p* > 0.05). Similarly, the presence of RAs did not affect submission rates (*p* = 0.53, Table 5). However, there was a significant difference in submission rates across the different assessment types. It was observed that Resimion assessments had a notably higher submission rates for both MCQs/case studies and weekly quizzes (95.4% and 93.5%) when compared to other assessment types (*p* = 0.002, Table 5).

**TABLE 5:** Percentages of students submitting assessments across several modules during the academic year 2022–23.

Assessment type	Submission rates 2022/23				
	All students	ND/LD only	RA excluding ND/LD	All RAs	Students without RAs
On campus, timed exam	Chem: 85.5% (*n* = 55)	83.3% (*n* = 6)	85.7% (*n* = 7)	84.6% (*n* = 13)	85.7% (*n* = 42)
	Haem: 87.7% (*n* = 65)	60.0% (*n* = 5)	88.9% (*n* = 9)	78.6% (*n* = 14)	90.2% (*n* = 51)
	SBOD: 83.2% (*n* = 155)	100% (*n* = 11)	75% (*n* = 28)	82.1% (*n* = 39)	83.6% (*n* = 116)
Online, 24 h exam	Blood science: 83.9% (*n* = 87)	88.9% (*n* = 9)	84.6% (*n* = 13)	81.8% (*n* = 22)	83.1% (*n* = 65)
Media clip	Blood Science: 70.1% (*n* = 87)	77.8% (*n* = 9)	84.6% (*n* = 13)	81.8% (*n* = 22)	66.2% (*n* = 65)
	Chem: 85.5% (*n* = 55)	83.3% (*n* = 6)	85.7% (*n* = 7)	84.6% (*n* = 13)	85.7% (*n* = 42)
Reflective coursework	SBOD: 83.9% (*n* = 155)	100% (*n* = 11)	85.7% (*n* = 28)	89.7% (*n* = 39)	82.0% (*n* = 116)
Written case study	SBOD: 85.8% (*n* = 155)	90.9% (*n* = 11)	89.3% (*n* = 28)	89.7% (*n* = 39)	84.5% (*n* = 116)
Resimion MCQs and case study	Haem: 95.4% (*n* = 65)	100% (*n* = 5)	88.9% (*n* = 9)	92.9% (*n* = 14)	96.1% (*n* = 51)
Weekly Resimion quizzes	SBOD: 93.5% (*n* = 155)	100% (*n* = 11)	89.3% (*n* = 28)	92.3% (*n* = 39)	94.0% (*n* = 116)

Total numbers of students in each group are shown in brackets. Chem, Clinical Biochemistry (FHEQ level 6 module). Haem, Haematology and Transfusion Science (FHEQ level 6 module). Blood Science, Blood Science (FHEQ level 5 module). SBOD, Studies in the Biology of Disease (FHEQ level 5 module). ND/LD, neurodiverse or specific learning disorders officially recorded by Disability Services. RAs, reasonable adjustments officially recorded by Disability Services.

### The Presence of Reasonable Adjustments Did Not Impact Student Outcome

The submissions presented here had a range of reasonable adjustments available to them in terms of extra time, grace periods, and extensions (Table 1). The available adjustments were made clear to students when the coursework was released. It is apparent from the data that no specific reasonable adjustment impacted student outcome or submission rate, regardless of RA status (Figures 3, 4; Table 5). The one exception to this was the timed Resimion case studies for Haematology and Transfusion Science, where students did use their additional allocated time (Figure 1), indicating that these adjustments are appropriate and enable students to gain comparable outcomes (Figure 4A). Finally, grace periods and extensions for assessments do not impact submission rates within this study, with Resimion assessments demonstrating the highest submission rates, whilst not eligible for any grace periods or extensions.

## Summary

In summary, this work successfully demonstrates good student engagement and positive feedback associated with the integration of Resimion into biomedical science teaching and assessment. Our data shows largely comparative outcomes through a range of assessment styles, in terms of overall achievement, with a better submission rate demonstrated for assessments completed using this novel approach. It seems that providing that assessment rules are clear in advance, the outcomes of students are comparable regardless of RA status (for Resimion-based and other assessment types), with only additional time for Resimion assessments shown to be important for eligible students. It is certainly clear from our data that no group of students are benefiting from any particular assessment type. Overall, this work demonstrates successful implementation of Resimion for SBL and gamification into biomedical science education.

## Discussion

### Benefits of Scenario-Based Learning and Gamification

There is a wealth of literature on the benefits of applied learning developing higher-level graduate skills, with “gamification” becoming an important topic in recent years [20, 21]. The integration of gamification has (predictably) provided the most success in computer science, representing 39% of the published literature. Comparatively, medicine, biology and psychology collectively account for 10% of gamification publications—a clear underperformer in the sector [22]. Our institution’s use of gamification increased significantly during the COVID-19 pandemic, for both assessment and within teaching, such as a replacement of lab classes which could not run due to social distancing, and Resimion was our software of choice. As discussed by Francis, Smith and Turner (2022) [23], virtual lab simulations were used to link theory and practice, closely mimicking the skills and learning objectives achieved in wet lab practicals, whilst providing an appropriate alternative when laboratory work was impractical. Furthermore, Resimion acted as an additional interactive method to maintain student interest and engagement in online tutorials. This concurs with other published reports, where improved student academic achievement, engagement and motivation were reported in 92.9% of studies examining gamification in education [21]. The anonymity of the software and the questions posed give the learner “permission to fail” without judgement from peers, and encourages a safe environment for learning. One challenge to using online tools during COVID-19 was the issue of digital poverty, with students having reduced access to campus resources. However, both Mentimeter and Resimion are used via a standard web browser, or mobile application, enabling students to access activities anywhere, on any internet-enabled device, removing many digital poverty barriers. Use of this tool was successful and well received by students. So much so that many of the changes have been maintained since returning to on-campus teaching.

This study demonstrates use of SBL can effectively be incorporated into many teaching types (online, face-to-face, asynchronous), in addition to assessments. Studies consistently highlight the need to use a variety of interactive learning activities in effective online teaching, including gamification, to capture student interest [24, 25]. Challenges through gamification can also entice students to continue playing [26]. A significant benefit of Resimion (alongside Mentimeter and Collaborate polling) was gaining real-time assessment of students understanding, which, as discussed by Neuwirth, Jovic and Mukherji (2021) [27], was challenging during COVID-19 when normal visual clues in a physical room could not be interpreted. Engagement data from Resimion was extremely useful when actual contact with students was so limited. It is widely believed in the literature that leveraging this data will provide further strategies to optimise learner engagement and outcome [28–30].

With so many changes over the last few years as a result of the COVID-19 pandemic (the rapid switch to online teaching, online exams, open-book exams, and loss of practical time) data from different years is not always comparable and it is difficult to isolate the impact of individual changes. One theme that has been consistent, however, is the positivity of students and staff toward using Resimion, with consistently good feedback from students. Use of Resimion also provides a method to support students with additional learning opportunities by providing asynchronous activities. This will become more important as we appreciate the longer-term impacts of students entering Higher Education with significant gaps in their education. A further benefit of using Resimion is the ability to monitor student engagement in real-time, providing an additional means to identify students who are struggling and need additional support.

### Authentic Assessments

“In this increasingly digital world, such [transferable] skills include digital literacy; opportunities are present to exploit this and diversify how students are assessed, ensuring a range of methodologies which allow students from all backgrounds and characteristics to demonstrate their learning and development. Assessments should be authentic, with real-world application to enhance employability skills and professional development” (QAA Benchmark Statements, 2023) [12].

Competency within the biomedical laboratory relies on knowledge, technical skills and attitude to work [31]. There is a need to provide authentic assessments, allowing students to face challenges in the safe setting of education. Traditional assessment styles of essays and examinations still have their place; however, these are increasingly complemented by other assessment formats. In this study we analysed data from a range of assessment types enabling a wider range of skills to be assessed. Many of these skills are essential for the workplace, making employable and professionally-directed graduates.

This study presents, for the first time, the integration of multiple Resimion scenario-based assessments into a university undergraduate programme. Assessments were disparate in style and content, covering all the biomedical science disciplines. These scenarios and case studies simulate real-world applications enabling the opportunity to apply the knowledge and understanding that students acquire during their degree.

Allowing students choice within assessments is also important and can enable experiences to be diverse and equally accessible to all. Within the Blood Science and the Clinical Biochemistry modules we designed the coursework assignment as a 5 min video recording that had a degree of student choice. Students often target the assignment towards their own interests, and select a subject that they, or a family member, have experienced. We have observed that students are willing to read widely about a subject if they have some ownership of the area, and often have increased engagement. The coursework was also designed to enable students to develop other key employability skills such as communication, following specific instructions, and presentation skills. Many students have commented that this has been a useful experience, and they appreciate having variation from some of the other traditional forms of assessment such as essays. Whilst initially some students find the challenge daunting, afterwards they can see how the skills they have gained will be useful in the future and feel proud of their achievements. Additionally, it gives students the chance to practice presentation skills in a safe environment, where they can have numerous attempts at recording their work. They also gain feedback on their communication skills, preparing them for an oral defence in their final year project and for future job interviews. Including these types of assessments within our study is reassuring to see that student engagement with assessments where they do not have choice are as good than those where some free choice is given. Providing students with a range of assessment types is essential to ensure that our graduates are fully equipped with the range of skills needed for successful employment.

### Impacts of COVID-19

COVID-19 and the associated lockdown fundamentally changed education, but also students. There has been a notable increase in student requests for reasonable adjustments within our institution, and it seems students are struggling across the sector. This raises the challenge of providing students with accessible assessments, whilst still fully addressing the learning outcomes of the module. There have even been occasions where students request the assessment type to be altered in order to address their reasonable adjustment. This obviously raises the issue of parity across assessment types and across the student cohort. Whilst our work demonstrates that students with RAs do not perform differently in each assessment type, it is apparent from the data that students are doing better in some assessments than others. For example, results for in-person exams was much lower when compared to other assessment types, which is perhaps not surprising given that our current cohort of students did not have the opportunity to undertake previous formal exams such as A-levels during COVID-19. Average exam marks are notably lower across multiple degree programmes at our university as the return to in-person exams has left students struggling, due to lack of prior experience with formal in-person examinations. In discussion with colleagues at other institutions, this has been observed across the sector (personal communication, 2023).

Student attendance/engagement at ours, in addition to other institutions across the sector remains an issue and declined after the COVID-19 pandemic. Although we have observed improved engagement of students with interactive tools like Mentimeter and Resimion, we still have a proportion of students who fail to attend or submit any work. For example, approximately 15% of students this year failed to pass the Resimion-based coursework in Studies in Biology of Disease, even though they could have unlimited attempts to complete this. Finding ways to engage these students remains a challenge.

Whilst we do have access to significant data in terms of student numbers (769 students in total), some of the modules analysed have a greater proportion of students with reasonable adjustments. These values range from 5% to 22% across the 8 cohorts we analysed, making the data from some modules more robust than others. The percentage of students with RAs has risen over recent years, with all cohorts this academic year having at least 20% of students with recorded disabilities. This may be the result of increasing awareness and support for learning differences and neurodiversity in particular. Indeed, the Office for Students reports an increase in disabilities from 7.3% of students in 2010–11 to 13.6% of all students in 2020–21. This increase includes a large rise in mental health disorders in particular (600% increase over this period), but also increases of 29% in cognitive or learning difficulties, 56% increase of students with multiple disabilities, and 700% increase rate of students with social or communication impairments [32]. It is also possible that we have significant numbers of students who are not formally diagnosed (particularly as neurodiverse/SpLD), with other studies similarly reporting that this is likely [33–35]. Informal discussions with some of our students also indicated that they consider themselves neurodiverse but do not have a formal diagnosis.

### Reasonable Adjustments

“All students should be offered learning and assessment opportunities that are equally accessible to them, using inclusive design wherever possible and by means of reasonable individual adjustments where necessary” (QAA Benchmark Statements, 2023) [12].

When integrating Resimion, one significant consideration was ensuring accessibility of Resimion for students with reasonable adjustments and specific learning needs. This is an increasingly important consideration given the rising numbers of students in Higher Education identifying as neurodivergent or having SpLDs [33]. When we initially trialled the software, students gave valuable feedback enabling additional accessibility features to be added. Subsequently, in collaboration with Resimion, we assessed the number of students utilising these self-modifiable accessibility features [36]. Some students on our modules with declared disabilities were also willing to discuss their experiences and use of these features, overall reporting positively.

We provide numerous opportunities for students to engage with Resimion before undertaking assessments (or multiple attempts in the case of “Studies in the Biology of Disease”) to enable students to familiarise themselves and experiment with accessibility features. This avoids “labelling” students, as discussed by Clouder et al. (2020) [35], and enables students who are unaware of their learning preferences/needs to experiment with options, and aid individuals who have chosen not to disclose a disability. The number of students with autism spectrum disorders is proposed to be underreported in many education institutions [34]. Therefore, many students will not have disabilities officially recorded, but would still benefit from accessibility features that they can self-modify. Additionally, the assessment format itself is more accessible than a traditional written assignment, focusing on decision-making and interpretation, without penalising for grammar and spelling. This is particularly challenging for students with specific learning needs [35], and those studying in a second language. Consequently, it is reassuring that we now have sufficient data to show that in addition to students with RAs reporting positively about their experience of using Resimion as a teaching and assessment tool, our data shows that they perform comparably to students without RAs (Figures 3, 4).

This work does, however, raise the question as to whether the presence of reasonable adjustments and extensions in particular, are actually good for the students. Whilst specific adjustments such as extra time and accessibility features aided students in our study, our data also demonstrated that students do not perform differently in the absence of grace periods and extensions. If these adjustments do not have a notable impact on outcome, is there a reason for them? Indeed, we see amongst some students that access to extensions is detrimental to those who struggle with time management. Constantly applying for extensions to coursework deadlines can result in further clashes and “bunching” of assessments later in the year and during the exam period.

### Limitations

Whilst our data show the positive impact of integrating Resimion into our teaching and assessment, it is important to acknowledge some of the challenges in a study such as this. In particular, the data presented is challenged by broad categories of RAs amongst our student population, e.g., “student has a mental health condition, challenge or disorder, such as depression, schizophrenia or anxiety.” This definition is broad enough to include vast differences in terms of challenges and support needs. Additionally, many students are listed simply as having “multiple disabilities” which can include both physical and learning differences (but not necessarily specified), or recorded as “an impairment, health condition or learning difference not listed above.” These wide-ranging categories and lack of information for some students posed challenges for us to accurately group students (and pose significant barriers for us to fully support these students in our teaching and assessment). Consequently, we grouped students with a recorded reasonable adjustment recorded in the “all RA” category, and further split students with a recorded specific learning difference, attention deficit (hyperactivity) disorder, or autism spectrum disorder within the “neurodiverse/specific learning difference” category. We did not include students with mental health conditions within this group, as there is controversy as to whether these conditions are classified as “neurodiverse,” even though there is noted co-occurrence [37]. These students with mental health conditions were included in the “all RAs” category, along with all other physical and unlisted/undeclared conditions.

## Recommendations

Every effort is made to ensure that assessment rules are clear and do not disadvantage particular groups of students. However, there needs to be a focus on evidence-based practice, especially in science education. If it is not clear that the changes we are making to submissions are benefitting students, we need to reassess their necessity.

There needs to be a diverse selection of education methods, along with assessment styles. Integration of technology into teaching is already underway and is becoming more pervasive. This is likely going to be a major advance in education, improving knowledge and skills in a form that is accessible to students regardless of neurodiversity or economic status [13]. However, the integration of technology must be tempered, as the occurrence of artificial Intelligence, such as ChatGPT, is likely going to make many assessments problematic (if not completely unusable). Programmes such as Resimion that assess knowledge and understanding simultaneously are likely to be the pathway to reduce the impact of artificial intelligence on assessment offences.

## Conclusion

In conclusion, this study has shown that staff and students can easily adapt to using Resimion, with the latter finding that it compliments and enhances their studies. Use of Resimion as an assessment tool has shown equivalent results for students with and without eligibility for adjustments, in addition to mapping to previous assessment results using more traditional methods. In addition, Resimion-based assessments are robust in the face of increasing levels of assessment offence, and ease of access to Artificial Intelligence services such as ChatGPT.

This work represents an advance in biomedical science because it demonstrates effective integration of Resimion into the pedagogy of an undergraduate Biomedical Science degree, with comparable outcomes amongst all student groups.

## Summary Table

### What Is Known About This Subject?

- Scenario-based learning and gamification has many advantages in comparison to traditional didactic teaching methods.
- Resimion is web-based and accessed from any internet-enabled device, removing many digital poverty barriers.
- Novel tools are needed to encourage engagement and ensure assessment integrity, however parity between these assessment types must be ensured.

### What This Work Adds

- Students with recorded adjustments (e.g., for disabilities, learning differences) obtain comparable results across a range of assessment types, including scenario-based activities using Resimion.
- Submission rates of assessments were comparable, if not improved, using scenario-based learning for assessment.
- Resimion is a promising integration of gamification into case-based learning in Biomedical Science, and was positively received by students.

## Data Availability

The raw data supporting the conclusion of this article will be made available by the authors, without undue reservation.
