# Supplementary material for: Gamification in Biomedical Science Education: The Successful Implementation of Resimion, a Scenario-Based Learning Tool
Source: Br J Biomed Sci. 2023 Oct 2;80:11756. doi: 10.3389/bjbs.2023.11756 (PMC10577182; doi:10.3389/bjbs.2023.11756)
Supplement: Supplementary file 1 [file DataSheet2.PDF]

Studies in the Biology of Disease 2022-23 – Coursework

Integrated case study: Mariana

The aim of the case study is to introduce you to the investigative nature of Biomedical Science. This will show how the individual disciplines integrate and aid the differential diagnosis of a patient. Your task is to interpret the data utilizing information learned during the module, ***backed up by information gleaned from your reading around the topic***. This will lead to a final diagnosis which will allow you to answer a specific question set.

You will need to think about the importance of the presenting symptoms, the initial results and any further investigations. You will need to think carefully on the differential diagnosis, how certain results rule out potential diagnosis or narrow the potential possibilities. Having made a diagnosis, you should think about potential treatment options and the pathophysiology that is represented in the case.

The following points are important to help you think about how to approach the case study:

Ensure you answer all questions asked. Do not ignore all or part of the questions.

Each question has a limit of 250 words. ***More than this will not be marked***.

Consider all results – ensure you discuss the results relevant to the question asked. For example, don't discuss the biochemistry results if asked about the haematology results. **Make sure you know which tests come under which discipline**.

Demonstrate your understanding of the underlying physiology that has gone awry, and you should continually refer to the patient, the presenting symptoms and results of the investigations.

Please use **UWE Harvard** as your referencing system. The reference section does not count towards the word count.

There is **no late submission** permitted for this assignment. However, there is a 5-day grace period.

Make sure you upload a **word document** (.doc or .docx).

Please upload your 1500-word write-up on blackboard before 2pm on Tuesday the 14th of March.

**Patient name: Mariana**	**Age: 19 years old**	**Gender: Female**
**Patient ethnicity: Asian British, of Chinese descent**		

Mariana is a 19-year-old undergraduate student who presented to her GP feeling tired and run down. This has lasted for a couple of weeks, and over the last few days Mariana has had a slightly raised temperature. She has lost a little weight recently (last few months). She has put her fatigue and weight loss down to the stress of university and irregular meals, whilst working long days keeping up with the new material. However, with the raise in temperature she agreed to take her friends advice and go see the GP. The GP agrees that it is likely stress related, and provides antibiotics if the condition gets worse.

Back in April, Mariana had spent 6-month's visiting family and friends in China, but also travelling throughout south east Asia including Cambodia, Malaysia, Thailand and Vietnam. In retrospect, she felt the stress from that visit may have contributed to her fatigue and weight loss. She took all required vaccinations for visiting the region, which were Diphtheria, Hepatitis A, Tetanus, and Typhoid. She did not receive further vaccinations and was on no other medication, apart from some aspirin in the last couple of days because of the raised temperature. She is a non-smoker and said she usually drinks moderately.

Table 1. Summary of travel history.

**Month**	**Notable comments from history**
**April**	In China. With friends and family. Passed out after drinking with friends at one point during the month.
**May**	In China. With friends and family. Also got a tattoo from a street parlour.
**June**	In Vietnam. Seemed to spend a lot of time with animals. Had serious stomach upsets at one point – thought to be food related.
**July**	In Cambodia. Went travelling through the Cambodian jungle. Reported eating "dodgy food".
**August**	In Thailand. Seemed to drink rather heavily this month (by own admission). Passed out multiple times.
**September**	In Malaysia. Much quieter. Little drinking occurred through this time. Had unprotected heterosexual intercourse with a fellow gap-year traveller.
[redacted]	
**December**	Mariana returns to the GP after 1 month.

The GP did a dipstick test on a urine sample that showed the presence of some protein and leukocyte esterase, so they sent both urine and faeces samples to microbiology. Further initial results were as follows:

Table 2. Haematology & Biochemistry blood results

**Investigation**	**Result (*normal range - female*)**
**Haematology**	
Red cell count (x 10^{12}/L)	4.2 (*3.8 – 5.8*)
Haemoglobin (g/L)	125 (*115 – 165*)
White cell count (x 10^{9}/L)	7.1 (*3.6 – 11.0*)
neutrophils	3.4 (*1.8 – 7.5*)
lymphocytes	3.1 (*1.0 – 4.0*)
monocytes	0.47 (*0.2 – 0.8*)
eosinophils	0.12 (*0.1 – 0.4*)
basophils	0.02 (*0.02 – 0.1*)
Platelets (x 10^{9}/L)	176 (*140 – 400*)
**Biochemistry**	
Blood Urea (mmol/L)	3.3 (*2.5 – 7.8*)
Serum creatinine (mmol/L)	85 (*45 – 84*)
Serum sodium (mmol/L)	140 (*133 – 146*)
Serum potassium (mmol/L)	3.6 (*3.5 – 5.3*)

Table 3. Microbiology Results

**Urine: Microscopy**	**Faeces: Microscopy**
WBC <5 per ml	No Cryptosporidium seen
RBC <5 per ml	No ova cysts or parasites seen in wet prep
Epithelial cells – moderate numbers	No ova cysts or parasites seen in concentrate
**Culture**	**Faeces: Culture**
No significant growth	No Salmonella, Shigella, Campylobacter, *E. coli* O157 or Vibrios isolated

Mariana returned to her GP one month later, as she was not getting any better. She reported that her urine was now a pale orange colour. The GP's examination now detected tenderness of lower abdomen and a number of bruises on her legs. On questioning she mentioned that she seemed to bruise very easily at the moment. The GP requested a further full blood count and liver function tests.

Table 4. Second Haematology and Biochemistry results

Investigation	Result (*normal range - female*)
**Haematology**	
Red cell count (x 10^{12}/L)	4.0 (*3.8 – 5.8*)
Haemoglobin (g/L)	123 (*115 – 165*)
White cell count (x 10^9/L)	8.1 (*3.6 – 11.0*)
neutrophils	2.9 (*1.8 – 7.5*)
lymphocytes	4.5 (*1.0 – 4.0*)
monocytes	0.62 (*0.2 – 0.8*)
eosinophils	0.10 (*0.1 – 0.4*)
basophils	0.02 (*0.02 – 0.1*)
Platelets (x 10^9/L)	138 (*140 – 400*)
Prothrombin Time (s)	20 (*10 – 14*)
Activated Partial Thromboplastin Time (s)	45 (*22 – 36*)
**Biochemistry**	
Serum Bilirubin (µmol/L)	50 (≤*21*)
Serum ALP (IU/L)	300 (*30 – 130*)
Serum AST (IU/L)	527 (≤*32*)
Serum ALT (IU/L)	650 (*10 – 35*)
Serum albumin (g/L)	37 (*35 – 50*)

On analysis of these haematology & biochemistry results, plus Mariana’s case history, the GP requested a further blood sample be taken and sent for Hepatitis B serology. The results of the serology are shown in the table below.

Table 5. Serology results

Hepatitis test	Test result
HBsAg	Positive
Anti-HBc	Positive
IgM Anti-HBc	Borderline
Anti-HBs	Negative

A sample was taken and sent for Group and Save so that Mariana's blood group was known in the event she would require blood components, results are given below for you to interpret.

	Forward group			Antibody Screen IAT 37°C		
	Anti-A	Anti-B	Anti-D	Cell I	Cell II	Cell II
Patient cells	0	4	4	0	0	0

Question Number	Question	Mark
1	**Haematology**<br>Detail the livers role in haemostasis. Explain how this relates to the results observed in Mariana.	20
2	**Biochemistry**<br>By comparing the laboratory investigations relative to the reference ranges, discuss all possible causes of the altered biochemical findings seen in Mariana's second set of results (**Table 4**).	20
3	**Immunology**<br>Discuss the serology results (**Table 5**) including your diagnosis of Mariana and your assessment as to whether she is infectious? What further tests would confirm the diagnosis and what treatments could you recommend to enhance her immune responses against this virus?	20
4	**Cellular pathology**<br>Describe the microscopic appearance of viral infected liver at the cellular level when stained with haematoxylin and eosin (H & E) on a tissue biopsy. Give examples of other histological stains that would be useful here, including expected results for Mariana.<br>What changes would you expect to observe in the liver at the tissue level of a patient with a chronic viral infection? What are the long-term implications for the patient if left untreated and how would this affect tissue function?	20
5	**Medical genetics**<br>Why is the Asian population at greater risk for hepatocellular carcinoma? Please include both genetic and non-genetic information in your answer.<br>Referring to current literature, summarise two key genetic pathways observed in hepatocellular carcinoma.	20
6	**Transfusion**<br>Do you think this patient requires a transfusion? Using current guidelines to support your answer, explain your reasoning. In your answer, detail the type of component and any relevant specifications to be considered.	20
