# Supplementary material for: Gamification in Biomedical Science Education: The Successful Implementation of Resimion, a Scenario-Based Learning Tool
Source: Br J Biomed Sci. 2023 Oct 2;80:11756. doi: 10.3389/bjbs.2023.11756 (PMC10577182; doi:10.3389/bjbs.2023.11756)
Supplement: Supplementary file 2 [file DataSheet3.PDF]

College of Health, Science and Society
Academic Year: 2022/2023
Assessment Period: 1

**Module Leader:**	**Dr Jonathon Hull**
**Module Code:**	**USSKAT-30-2**
**Module Title:**	**Studies in the Biology of Disease**
**Examination Start Time:**	**09.30**
**Duration:**	**3-hour exam**

ON CAMPUS EXAM

Standard materials required:

Examination Answer Booklet	Yes
Multiple Choice Answer Sheet	No
Type of Graph Paper	None
Number of graph paper sheets per student	0

Additional materials required for this examination:
To be supplied by UWE Bristol
Please list any additional material and whether or not they need to be collected:

No additional materials are required to be supplied by UWE

To be supplied by the student
Please list any additional material and whether or not they need to be collected:

Students are permitted to bring in an annotated copy of the case study (4 pages).
This must be collected at the end of the exam.

University approved calculator (non-programmable)	Yes
Students permitted to keep this examination paper	No

Students are not permitted to turn this page over until the examination starts

Patient name: Christina	Age: 50 years old	Gender: Female
**Patient ethnicity: European British, of Cypriot descent**		

Fifty-year-old Christina was admitted to the Avon Orthopaedic Centre for an emergency total hip replacement operation after she fell down the stairs at home and fractured the top of her left femoral shaft.

During the pre-op assessment she seemed a little confused and disorientated. Fortunately, her daughter had accompanied her to the hospital and was able to tell the Registrar that her mother had complained of feeling tired and weak for a little while. The daughter also said that her mother was often “a little forgetful”. Whenever she had suggested to her mother that she saw her GP to check things over, Christina had said not to fuss and that it was all “stuff and nonsense”.

The Registrar then ascertained as reasonable a medical history as she could from the daughter. She reported that her mother was not on any medication other than some tablets she had from the chemist to help with her vertigo. Furthermore, she had not had any major illness that she was aware of, although she had been in hospital 5 years ago following a road traffic accident. She could remember that her mother had required a blood transfusion, but off hand could not remember how many units she had required.

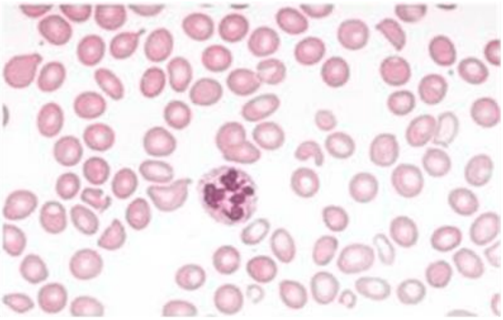

Figure 1: Christina Markides blood film. Blood film showing macrocytic red cells with hypersegmented neutrophils

The Registrar took a sample for a full blood count (**Table 1**) and film (**Figure 1**) and also for a Group and Save as a blood transfusion will be required during the hip operation. The blood film reported macrocytic red cells with hyper-segmented neutrophils (**Figure 1**).

Table 1. Initial Haematology results

Investigation	Result (*reference range - female*)
Red cell count (x 10^{12}/L)	3.0 (*3.8 – 5.8*)
Haemoglobin (g/L)	100 (*115 – 165*)
Haematocrit (L/L)	0.34 (*0.37 – 0.47*)
MCV (fl or μm^3)	105 (*80 – 100*)
MCH (pg/cell)	33 (*27 – 32*)
White cell count (x 10^9/L)	5.6 (*3.6 – 11.0*)
Platelet count (x 10^9/L)	197 (*140 – 400*)

On receiving the results, the Registrar asked Christina's daughter if she was aware if any other members of her family had ever presented with similar symptoms. She replied that although her parents had come over from Cyprus in the 1970s, they travelled back frequently and kept in regular contact with their extended family. As far as she was aware, there was no family history of anaemia. In delving further into Christina's background, her daughter mentioned that Christina was a strict vegetarian. The Registrar took further blood samples to determine serum B12 and folate levels, and to check for the presence of autoantibodies.

Table 2. Additional Haematological and Immunological results

**Investigation**	**Result (*normal range - female*)**
Serum folate (µg/L)	2.8 (*2.4 – 17.5*)
Serum B12 (ng/L)	64 (*197 – 771*)
Serum ferritin (µg/L)	127 (*10 – 300*)
Intrinsic factor antibodies	negative
Parietal cell antibodies	positive

One month later, the anaemia has not resolved. A repeat panel was performed in case another transfusion was required (**Panel 2**).

Product	Lot no.	Product	Lot no.	Product	Lot no.
ID Panel in Alsevers	Rxxxxxx	ID Panel in Papainised in Alsevers	Rxxxxxx	ID Panel LISP	Rxxxxxx
ID Panel in CellStab	Rxxxxxx	ID Panel in Papainised in CellStab	Rxxxxxx	Expiry dd/mm/yyyy	
ID Panel in CellMedia	Rxxxxxx	ID Panel in Papainised in CellMedia	Rxxxxxx		

Patient's Name DOB		Conclusion

Unless otherwise indicated, all cells are positive for Kp^b and Lu^a and negative for Wr^a and Co^b

	Rh	C	D	E	c	e	C^w	M	N	S	s	P1	Lu^a	K	k	Kp^a	Le^a	Le^b	Fy^a	Fy^b	Jk^a	Jk^b	Other	Sal 18°C	IAT 37°C	Enz 37°C		
1	$R_1^wR_1$	+	+	0	0	+	+	0	+	0	+	0	0	0	+	0	0	+	0	+	0	+			0			
2	R_1R_1	+	+	0	0	+	0	+	0	+	0	0	0	+	+	0	0	0	+	0	+	0			0			
3	R_2R_2	0	+	+	+	0	0	0	+	0	+	3	0	0	+	0	+	0	+	0	0	+			4			
4	r'r	+	0	0	+	+	0	+	0	+	0	0	0	0	+	0	0	+	+	0	+	0			0			
5	r''r	0	0	+	+	+	0	+	0	+	0	2	0	0	+	0	0	+	0	+	+	0			4			
6	rr	0	0	0	+	+	0	+	0	0	+	1	0	+	0	0	0	+	0	+	0	+			0			
7	rr	0	0	0	+	+	0	0	+	0	+	4	0	+	+	0	+	0	+	0	0	+	Co^b+		0			
8	rr	0	0	0	+	+	0	0	+	0	+	0	0	0	+	+	0	+	+	0	+	0			0			
9	rr	0	0	0	+	+	0	0	+	+	0	3	0	0	+	0	0	+	+	0	0	+			0			
10	rr	0	0	0	+	+	0	0	+	0	+	0	+	0	+	0	+	0	0	+	+	0			0			
Auto																									0			
R1r vs Wk anti-D																									3			

Reagent							DAT profile	Anti-IgG	IgA	IgM	C3c	C3d	Ctrl
Lot no.							Result						

Panel 1: Antibody ID panel for Christina attending the clinic with a fractured hip.

Product	Lot no.	Product	Lot no.	Product	Lot no.
ID Panel in Alsevers	Rxxxxxx	ID Panel in Papainised in Alsevers	Rxxxxxx	ID Panel LISP	Rxxxxxx
ID Panel in CellStab	Rxxxxxx	ID Panel in Papainised in CellStab	Rxxxxxx	Expiry dd/mm/yyyy	
ID Panel in CellMedia	Rxxxxxx	ID Panel in Papainised in CellMedia	Rxxxxxx		

Patient's Name<br>DOB		Conclusion

Unless otherwise indicated, all cells are positive for Kp^b and Lu^a and negative for Wr^a and Co^b

	Rh	C	D	E	c	e	C^w	M	N	S	s	P1	Lu^a	K	k	Kp^a	Le^a	Le^b	Fy^a	Fy^b	Jk^a	Jk^b	Other	Sal 18°C	IAT 37°C	Enz 37°C		
1	$R_1^wR_1$	+	+	0	0	+	+	0	+	0	+	0	0	0	+	0	0	+	0	+	0	+			0			
2	R_1R_1	+	+	0	0	+	0	+	0	+	0	0	0	+	+	0	0	0	+	0	+	0			4			
3	R_2R_2	0	+	+	+	0	0	0	+	0	+	3	0	0	+	0	+	0	+	0	0	+			4			
4	r'r	+	0	0	+	+	0	+	0	+	0	0	0	0	+	0	0	+	+	0	+	0			4			
5	r''r	0	0	+	+	+	0	+	0	+	0	2	0	0	+	0	0	+	0	+	+	0			4			
6	rr	0	0	0	+	+	0	+	0	0	+	1	0	+	0	0	0	+	0	+	0	+			0			
7	rr	0	0	0	+	+	0	0	+	0	+	4	0	+	+	0	+	0	+	0	0	+	Co^b+		0			
8	rr	0	0	0	+	+	0	0	+	0	+	0	0	0	+	+	0	+	+	0	+	0			4			
9	rr	0	0	0	+	+	0	0	+	+	0	3	0	0	+	0	0	+	+	0	0	+			0			
10	rr	0	0	0	+	+	0	0	+	0	+	0	+	0	+	0	+	0	0	+	+	0			4			
Auto																									0			
R1r vs Wk anti-D																									3			

Reagent							DAT profile	Anti-IgG	IgA	IgM	C3c	C3d	Ctrl
Lot no.							Result						

Panel 2: Antibody ID panel for Christina one month after attending the clinic with a fractured hip.

Instructions to Candidates:

Taking all of the results into consideration, please answer ALL the following questions

Question Number	Question	Marks (%)
1	**Medical Genetics**<br>What are the possible **genetic** causes of anaemia in someone presenting with Christina's history? You should ***detail*** the mechanisms by which anaemia results in each case.<br>Can you rule out any of these possible causes on the basis of the initial results obtained from the tests requested by the Registrar, and the answers given by Christina's daughter in response to the Registrar's additional questions? Justify your decisions.	20
2	**Transfusion**<br>Using **Panel 1**, identify any **allo**-antibody present in the patient's plasma, and **explain** your reasoning. How have you eliminated the presence of other antibody specificities?<br>One month later, Christina's anaemia has not resolved. What has changed and show your reasoning (**Panel 2**)? What is the future significance of this new finding to our patient?	20
3	**Haematology**<br>Describe the mechanisms that might cause the marrow to produce macrocytes and hypersegmented neutrophils. Include in your answer how asynchrony between nucleus and cytoplasm occurs.	20
4	**Clinical Biochemistry**<br>Christina broke her hip falling down the stairs. What conditions might make an older woman's bones brittle and could have contributed to the break? Explain the mechanisms by which these disorders give rise to brittle bones.	20
5	**Immunology**<br>Taking into account the additional haematological and immunological results (**Table 2**), suggest a final diagnosis. Explain how you have reached this conclusion.<br>Detail the pathophysiology represented by the immunology results here.	20

END OF QUESTION PAPER
