# Supplementary material for: Gamification in Biomedical Science Education: The Successful Implementation of Resimion, a Scenario-Based Learning Tool
Source: Br J Biomed Sci. 2023 Oct 2;80:11756. doi: 10.3389/bjbs.2023.11756 (PMC10577182; doi:10.3389/bjbs.2023.11756)
Supplement: Supplementary file 3 [file DataSheet1.PDF]

Studies in the Biology of Disease – Coursework December 2022

IBMS Journal Based Learning Style Activity.

This activity is designed to mimic the Journal Based Learning (JBL) Continuing Professional Development (CPD) activity that the IBMS offers its members within the website and their magazine. Jon showed you some examples of these in the first lecture, they have also been available on blackboard since the beginning of the module. In the first lecture one of the topics we covered was an introduction to the expectations of quality placed on clinical labs relating to ISO15189. So, for this activity you will be reading a paper about quality management systems (QMS) which builds on that introduction. You may find the paper a little heavy going, that is to be expected, but the JBL approach requires that you read the paper carefully to answer the first part of the activity – the true/false statements.

In the table below, there are 20 statements you need to determine if they are true or false. I have set them out in the order of the paper to make this first attempt at this type of activity a little easier for you. Some of the questions may be about information in one of the tables.

<u>You need to access this paper</u>: Allen, L.C., (2013) Role of a quality management system in improving patient safety—laboratory aspects. *Clinical Biochemistry*, 46(13-14), pp.1187-1193. https://doi.org/10.1016/j.clinbiochem.2013.04.028

If you click on this link and then log in through UWE you will be able to access it.

Question	Statement	True or False<br>(Insert your answer)
1	Canada participates in ISO through the Standards Council of North America	
2	Quality management is all the activities undertaken to direct, control and co-ordinate quality	
3	QMS is a network of interrelated and interconnected processes	
4	Patient safety aims to maximise the incidence and impact of adverse events	
5	A study in Belgium used 29 different questionnaires to assess the impact of ISO certification	
6	Newfoundland was the territory that had the poorest record of laboratory related patient safety issues in Canada in 2005	
7	The lack of uniform testing procedures was deemed to raise the risk of misinterpretation	
8	In Manitoba in 2012 there were 10 critical incidents	
9	ISO15189 requires document control so that only the current version is accessible	
10	Laboratory professionals should not provide advice on the choice of test	
11	Staff can audit their own work	

12	Lab equipment must have its performance verified on installation	
13	Samples that are not properly labelled should be rejected by the lab	
14	A process for dealing with “non-conforming results” must be in place	
15	Staffing levels need to allow capacity for ensuring QMS is followed	
16	Equipment can be run outside its optimal environmental conditions	
17	Small equipment such as pipettes are exempt from maintenance requirements	
18	In-house methods are not permitted by ISO certification	
19	Inter-instrument comparison across all platforms is required	
20	Ontario labs reported an average conformance of 96.77% in 2010-11	

Longer Questions (250 words each)

1: Using published literature to support your answer; how well do you think you would meet the standards expected in the clinical laboratory sector under ISO15189? How could you improve for practicals in the second semester and moving forward into your project next year? You will want to interact with published literature in this answer.

2: How could you apply the principles of QMS to your own approaches to coursework assignments? Use the material in table 2 come up with parallels to undertaking academic work.
